# Chloroplast genome of *Aconitum barbatum* var.* puberulum* (Ranunculaceae) derived from CCS reads using the PacBio *RS* platform

**DOI:** 10.3389/fpls.2015.00042

**Published:** 2015-02-06

**Authors:** Xiaochen Chen, Qiushi Li, Ying Li, Jun Qian, Jianping Han

**Affiliations:** Center for Computational Biology and Bioinformatics, Institute of Medicinal Plant Development, Chinese Academy of Medical Sciences and Peking Union Medical CollegeBeijing, China

**Keywords:** chloroplast genome, *Aconitum*, circular consensus sequencing, PacBio *RS*, the third generation sequencing

## Abstract

The chloroplast genome (cp genome) of *Aconitum barbatum* var.* puberulum* was sequenced using the third-generation sequencing platform based on the single-molecule real-time (SMRT) sequencing approach. To our knowledge, this is the first reported complete cp genome of *Aconitum*, and we anticipate that it will have great value for phylogenetic studies of the Ranunculaceae family. In total, 23,498 CCS reads and 20,685,462 base pairs were generated, the mean read length was 880 bp, and the longest read was 2,261 bp. Genome coverage of 100% was achieved with a mean coverage of 132× and no gaps. The accuracy of the assembled genome is 99.973%; the assembly was validated using Sanger sequencing of six selected genes from the cp genome. The complete cp genome of *A. barbatum* var.* puberulum* is 156,749 bp in length, including a large single-copy region of 87,630 bp and a small single-copy region of 16,941 bp separated by two inverted repeats of 26,089 bp. The cp genome contains 130 genes, including 84 protein-coding genes, 34 tRNA genes and eight rRNA genes. Four forward, five inverted and eight tandem repeats were identified. According to the SSR analysis, the longest poly structure is a 20-T repeat. Our results presented in this paper will facilitate the phylogenetic studies and molecular authentication on *Aconitum*.

## INTRODUCTION

*Aconitum barbatum* var.* puberulum* (Niubian) belongs to the *Aconitum* subgenus *Lycoctonum* (Ranunculaceae) and most species of *Lycoctonum* are low-temperature resistant. However, aconitine is a kind of highly toxic alkaloid, which mainly exists in the plants of *Aconitum*. Identification and phylogeny studies of *Aconitum* and the Ranunculaceae family thus are particularly important (Xiao et al., 2005). He et al. (2010) applied the chloroplast genome (cp genome) intergenic region *psbA-trnH* as a barcode to identify 19 species in *Aconitum*, and Johansson (Johansson, 1995) used chloroplast DNA restriction site variation among 31 genera of the Ranunculaceae to conduct phylogenetic analyses. However, more in-depth studies of the cp genome are needed.

Chloroplasts possess their own genome and genetic system, which plays an important role in photosynthesis. The first chloroplast genome to be sequenced was that of *Nicotiana tabacum*, which heralded a new age of chloroplast studies in photobiology, phylogenetic biology, evolutionary biology and even chloroplast genetic engineering (Shinozaki et al., 1986; Hiratsuka et al., 1989; Daniell et al., 1998; Pfannschmidt et al., 1999; Moore et al., 2010; Wu et al., 2012). Some researchers (Chen et al., 2014; Li et al., 2014b) advocated the cp genome as a new DNA barcode to distinguish closely related plants. The typical cp genome structure of higher plants is circular with a length of 120–160 kb, containing approximately 130 genes (Sugiura, 1992). Two inverted repeats (IRs), a large single-copy region (LSC), and a small single-copy region (SSC) constitute the complete cp genome (Zhang et al., 2012). With the development of the next-generation sequencing technology, increasing numbers of species have been sequenced, including duckweed, palm, and others (Jordan et al., 1996; Uthaipaisanwong et al., 2012). Although the interest in the cp genome has increased in the past few decades, with 486 complete cp genome sequences deposited in GenBank (By 2014-7-6), there are still challenges and opportunities to develop a simple and rapid method for sequencing cp genomes. One common strategy is the use of a complete set of universal primers to amplify an entire cp genome and then perform the sequencing (Dong et al., 2013). Another frequently used strategy is “whole-genome sequencing”, which uses the total genome DNA to recover the cp genome through massively parallel sequencing (McPherson et al., 2013). This strategy is quite simple and effective, particularly as the cost of high-throughput sequencing decreases. In the present study, we used purified chloroplast DNA as the template for sequencing with the aim of developing a practical strategy involving the use of multiple samples to sequence the cp genome on the PacBio *RS* platform.

The third-generation PacBio system is based on the single-molecule real-time (SMRT) sequencing approach (Eid et al., 2009). Second-generation sequencing introduced a novel, rapid method for whole-genome sequencing (Mardis, 2008a,b; Metzker, 2009; Gilles et al., 2011; Kircher et al., 2011). In comparison, the SMRT approach requires no amplification, produces less compositional bias (Schadt et al., 2010), reduces the time required from sample to sequence (Chin et al., 2011; Rasko et al., 2011) and reduces the costs (Rusk, 2009). However, the main advantage of third-generation sequencing is the long read length, which was reported to be as long as 3,000 bp on average, and some reads might be 20,000 bp or longer. The long read length provides an important benefit for *de novo* assemblies, it allows the discovery of large structural variants, and it provides accurate microsatellite lengths, sensitive SNP detection and haplotype blocks (Metzker, 2009; Roberts et al., 2013; Li et al., 2014a). Because of the unique circular structure of the cp genome, the four junctions between the inverted regions and the single-copy regions have hampered our ability to provide accurate cp genome assemblies. However, the long reads somehow will promote and heighten the accuracy of the assembly (Bashir et al., 2012; Chin et al., 2013). SMRT sequencing combined with circular consensus sequencing (CCS) is thought to be an effective approach. This sequencing method provides multiple reads of individual templates, resulting in a higher per-base sequencing accuracy and a reduced error rate. A PacBio-only assembly could be completed without the need to construct specialized fosmid libraries or other similar assemblies using second-generation sequencing technologies. We sought to investigate whether third-generation sequencing could be used for rapid sequencing of whole cp genomes and eliminate the need to fill in the gaps that exist in the assembled genome.

In the present study, we report the completed cp genome of *A. barbatum* var.* puberulum*. To our knowledge, this is the first completed cp genome of *Aconitum* using the third-generation sequencing platform. Our results demonstrate that the SMRT CCS sequencing strategy is a viable option for rapidly sequencing cp genomes.

## MATERIALS AND METHODS

### CHLOROPLAST DNA ISOLATION, SEQUENCING, ASSEMBLY, AND VALIDATION

Fresh leaves were collected from Donglingshan Mountain, Beijing. Total cpDNA was extracted from approximately 100 g fresh leaves using a sucrose gradient centrifugation method that was described by Li et al. (2012). A total of 700 ng cp genomic DNA was sheared to a target size of 2 kb in an AFA clear mini-tube using a Covaris S2-focused ultrasonicator (Covaris Inc.) to construct the libraries according to the Pacific Biosciences SMRT Sequencing instruction manual. A 0.6X volume of pre-washed AMPure XP magnetic beads was added to the solution of sheared DNA. After concentrating the DNA, an Agilent 2100 and a Qubit fluorometer were used to perform qualitative and quantitative analyses. The samples were incubated at 25°C for 15 min to end-repair the DNA using the PacBio DNA Template Pre Kit 2.0. Then the end-repaired DNA was purified by adding a 0.6X volume of pre-washed AMPure XP magnetic beads. Blunt ligation was performed to obtain the SMRTbell^TM^ Templates, followed by the addition of exonuclease to remove failed ligation products. The SMRTbell^TM^ Templates were then purified in two steps. Before annealing the sequencing primer and binding polymerase to the SMRTbell templates, an Agilent 2100 and a Qubit fluorometer were used to perform the qualitative and quantitative analysis. PacBio DNA/polymerase Binding Kit 2.0 was used to anneal and bind the SMRTbell^TM^ Templates. Two SMRT cells were used with C2 chemistry to sequence the SMRT-bell^TM^ library. Two 45-min windows were captured for sequencing the chloroplast genome. After the CCS reads were derived from the multiple alignments of sub-reads, a quality control step was performed for the downstream assembly: SMRT Portal software (v2.0.0) was used to filter out the sequencing adapters and low-quality sequences (default parameters: sub-read length ≥ 50 bp; polymerase read quality ≥ 0.75; polymerase read length ≥ 50 bp; Li et al., 2014a). The reads were then used to assemble the chloroplast genome according to the strategy described in Qian et al. (2013). First, a workflow was designed to assemble the chloroplast genome: algorithms for greedy assembly, mapping, and consensus calling were used sequentially. Second, BLAST was used to compare the sequences from the greedy workflow, and the results of the alignment were used to construct the raw cp genome. The reads were mapped to the raw cp genome using the BWA tool, and the final cp genome sequence was generated using CAP3-based consensus calling (Altschul et al., 1997; Huang and Madan, 1999; Li and Durbin, 2010). To verify the genome sequence, PCR-based conventional Sanger sequencing was performed on six chloroplast genes (*cemA, psbB, psbC, rpoA, rpoC1, and rps4*; Cronn et al., 2008). The four junctions between the single-copy regions and the IRs were validated using PCR. The amplified sequences and the SMRT sequencing-based reads were aligned using Mega 5.2.2 (Tamura et al., 2011).

### GENOME ANNOTATION AND CODON USAGE

The cp genome was annotated using the program DOGMA (Wyman et al., 2004; default parameters: the percent identity cutoff for protein coding genes=60%, the percent identity cutoff for RNAs = 80%, the *E*-value = 1e-5 and the number of blast hits to return = 5.), and the position of each gene was determined using a blast method with the complete cp genome sequence of *Ranunculus macranthus* (GenBank Acc. No. NC_008796) as a reference sequence. Manual corrections for start and stop codons and for intron/exon boundaries were performed by referencing the Chloroplast Genome Database (ChloroplastDB; Cui et al., 2006). The tRNA genes were identified using DOGMA and tRNAscan-SE (Schattner et al., 2005). The circular cp genome map of *A. barbatum* var.* puberulum* was drawn using the OrganellarGenome DRAW tool (ORDRAW; Lohse et al., 2007). Codon usage and GC content were analyzed by Mega 5.2.2.

### REPEAT ANALYSIS

REPuter (Kurtz et al., 2001) was used to assess both direct and IRs according to the following criteria: cutoff *n* ≥ 30 bp and 90% sequence identities (Hamming distance equal to 3). Tandem Repeats Finder (TRF) v4.04 (Benson, 1999) was used to analyze tandem repeats with the settings reported by Nie et al. (2012). Simple sequence repeats (SSRs) were detected using MISA (http://pgrc.ipk-gatersleben.de/misa/), with thresholds of eight, four and three units for mono-, di-, and trinucleotide SSRs and tetra-, penta-, and hexanucleotide SSRs, respectively.

## RESULTS

### PacBio *RS* OUTPUT AND GENOME VALIDATION

Quantitative analysis using an Agilent 2100 showed that the average length of the sheared DNA fragments was approximately 1 kb. In total, 23,498 CCS reads and 20,685,462 base pairs were generated, the mean read length was 880 bp, and the longest read was 2,261 bp. Genome coverage of 100% was achieved with a mean coverage of 132× and no gaps. Detailed information is listed in **Table 1**. Six conserved genes with poly-structures (*cemA, psbB, psbC, rpoA, rpoC1*,* and rps4*) and four junction regions were validated using Sanger sequencing. The validated genes amounted to 7,341 bp, and a comparison of the assembled cp genome sequence with the Sanger sequencing results in these regions showed two mismatches in *psbB*, giving an error rate of 0.027%.

**Table 1:** Summary of the sequencing data for PacBio SMRT.

	Raw data		Clean data
Number of reads	300,582		23,498
Number of nucleotides (bp)	673,594,247		20,685,462
Longest read length (bp)	11,914		2,261
Mean read length (bp)	2,241		880
Genome coverage %		100%	
Average depth of coverage		132×	
Number of contigs		1	
Whole cp genome length (bp)		156,749	
Run time		45 min×2	
Total DNA requirements (ng)		700	

### GENOME FEATURES

**FIGURE 1:**
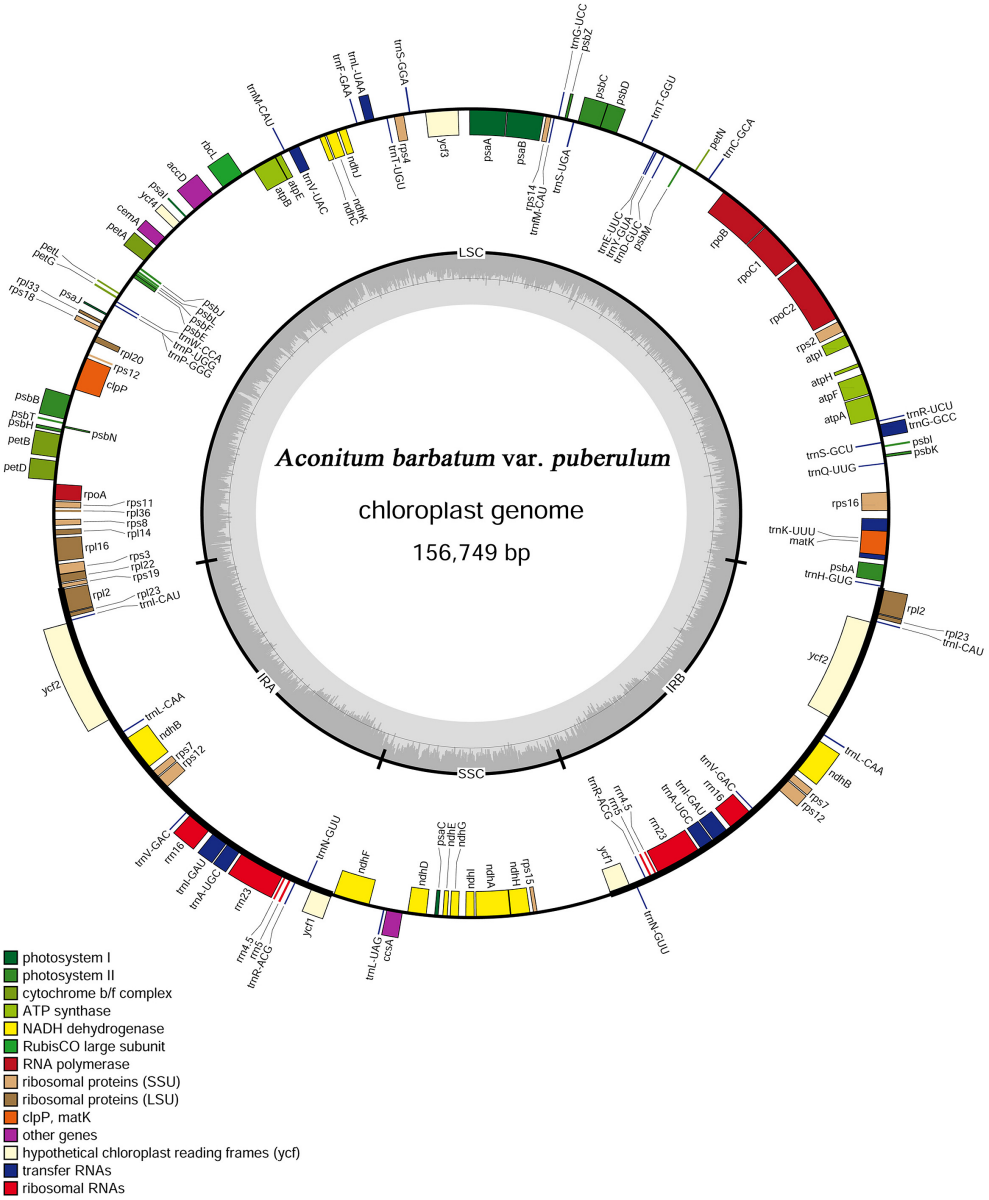
**Gene map of the *Aconitum barbatum* var.* puberulum* chloroplast genome**.

The complete cp genome of *A. barbatum* var.* puberulum* (GenBank acc. No. KC844054) was 156,749 bp in length with the common quadripartite structure found in most land plants (**Figure 1**), which included a LSC of 87,630 bp and a SSC of 16,941 bp separated by two IRs of 26,089 bp. In accordance with most chloroplast genomes, the nucleotide composition of* A. barbatum* var.* puberulum* was biased toward A+T (Sato et al., 1999; Nie et al., 2012; Pan et al., 2012; Yi and Kim, 2012). Overall, the *A. barbatum* var.* puberulum* cp genome A+T content was 61.3%, and the LSC and SSC regions (63.9 and 67.3%, respectively) were higher in A+T content than the IR regions (57.0%; **Table 2**).

**Table 2:** General information of the *Aconitum barbatum* var.* puberulum* chloroplast genome.

Length and GC content of the four regions				

	**Whole genome**	**LSC region**	**SSC region**	**IR region**
Length (bp)	156,749	87,630	16,941	26,089
GC content (%)	38.7	36.1	32.7	43.0

**Number of total genes and intron-containing genes**				

**Protein-coding**				

	**Total genes**	**regions**	**tRNA**	**rRNA**

Number of genes	130	84	31	4
Number of intron-containing	18	12	6	0

The *A. barbatum* var.* puberulum* cp genome contained 84 protein-coding regions, including seven genes (*rpl2, rpl23, ycf2, ndhB, rps7, rps12*,* and ycf1*) that were duplicated in the IR regions. In total, 31 unique tRNA genes (including seven tRNA genes located in the IR regions, *trnI-CAU, trnL-CAA, trnV-GAC, trnI-GAU, trnA-UGC, trnR-ACG* and* trnN-GUU*) were distributed throughout the cp genome, and four rRNA genes were duplicated in the IR regions. In summary, the cp genome of *A. barbatum* var.* puberulum* contained 130 genes, 18 of which were intron-containing genes (**Table 3**). Three of the intron-containing genes (*ycf3, clpP*,* and rps12*) had two introns, and the other 15 had only one intron. The 5′ end of *rps12* was located in the LSC region, and the 3′ end was located in the IR region, which caused trans-splicing in *rps12*. In addition, the sequence of *psbD* in the cp genome of *A. barbatum* var.* puberulum* differed from that in the reference sequence from *R. macranthus* (GenBank: NC_008796), which was found to be complementary. Moreover, *infA* was not present in the cp genome of *A. barbatum* var.* puberulum*; this gene codes for translation initiation factor 1 and is suspected to be an example of chloroplast-to-nucleus gene transfer (Millen et al., 2001). The codon usage and codon-anticodon recognition pattern of the cp genome are summarized in **Table 4**. The 31 unique tRNA genes included codons for all 20 amino acids necessary for biosynthesis. Leucine and serine (three of the 31, respectively) were the two most common amino acids represented by the codons of the tRNA in the cp genome.

**Table 3:** The genes with introns in the *A. barbatum* var.* puberulum* chloroplast genome and the length of the exons and introns.

Gene	Location	Exon (bp)	Intron (bp)	Exon (bp)	Intron (bp)	Exon (bp)
*trnK-UUU*	LSC	35	2528	37		
*rps16*	LSC	251	887	40		
*trnG-GCC*	LSC	23	738	48		
*atpF*	LSC	407	779	124		
*rpoC1*	LSC	1612	758	428		
*ycf3*	LSC	155	746	240	735	124
*trnL-UAA*	LSC	35	495	50		
*trnV-UAC*	LSC	37	597	39		
*clpP*	LSC	246	663	289	833	71
*petB*	LSC	6	948	489		
*petD*	LSC	8	704	496		
*rpl16*	LSC	399	1069	9		
*rpl2*	IR	434	667	391		
*ndhB*	IR	756	702	777		
*trnI-GAU*	IR	42	937	35		
*trnA-UGC*	IR	38	802	35		
*ndhA*	SSC	539	1003	553		
*rps12*	LSC	114		232	544	26

**Table 4:** The codon–anticodon recognition pattern and codon usage for the *A. barbatum* var.* puberulum* chloroplast genome.

Animo acid	Codon	No.	RSCU	tRNA	Animo acid	Codon	No.	RSCU	tRNA
Phe	UUU	834	1.23		Tyr	UAU	714	1.59	
Phe	UUC	519	0.77	*trnF-GAA*	Tyr	UAC	185	0.41	*trnY-GUA*
Leu	UUA	765	1.77	*trnL-UAA*	Stop	UAA	36	1.29	
Leu	UUG	554	1.29	*trnL-CAA*	Stop	UAG	26	0.93	
Leu	CUU	542	1.26		His	CAU	488	1.52	
Leu	CUC	192	0.45		His	CAC	153	0.48	*trnH-GUG*
Leu	CUA	356	0.83	*trnL-UAG*	Gln	CAA	640	1.5	*trnQ-UUC*
Leu	CUG	177	0.41		Gln	CAG	215	0.5	
Ile	AUU	1017	1.46		Asn	AAU	901	1.54	
Ile	AUC	430	0.62	*trnI-GAU*	Asn	AAC	267	0.46	*trnN-GUU*
Ile	AUA	646	0.93	*trnI-CAU*	Lys	AAA	889	1.44	*trnK-UUU*
				*trn(f)M-CAU*,					
Met	AUG	602	1	*trnM-CAU*	Lys	AAG	345	0.56	
Val	GUU	507	1.45		Asp	GAU	809	1.6	
Val	GUC	157	0.45	*trnV-GAC*	Asp	GAC	200	0.4	*trnD-GUC*
Val	GUA	527	1.51	*trnV-UAC*	Glu	GAA	924	1.45	*trnE-UUC*
Val	GUG	208	0.59		Glu	GAG	348	0.55	
Ser	UCU	538	1.67		Cys	UGU	220	1.5	
Ser	UCC	335	1.04	*trnS-GGA*	Cys	UGC	73	0.5	*trnC-GCA*
Ser	UCA	390	1.21	*trnS-UGA*	Stop	UGA	22	0.79	
Ser	UCG	191	0.59		Trp	UGG	432	1	*trnW-CCA*
Pro	CCU	406	1.52		Arg	CGU	348	1.38	*trnR-ACG*
Pro	CCC	214	0.8	*trnP-GGG*	Arg	CGC	86	0.34	
Pro	CCA	311	1.16	*trnP-UGG*	Arg	CGA	346	1.38	
Pro	CCG	138	0.52		Arg	CGG	114	0.45	
Thr	ACU	502	1.57		Arg	AGA	451	1.79	*trnR-UCU*
Thr	ACC	240	0.75	*trnT-GGU*	Arg	AGG	163	0.65	
Thr	ACA	393	1.23	*trnT-UGU*	Ser	AGU	372	1.15	
Thr	ACG	140	0.44		Ser	AGC	108	0.34	*trnS-GCU*
Ala	GCU	589	1.73		Gly	GGU	592	1.36	
Ala	GCC	220	0.65		Gly	GGC	177	0.41	*trnG-GCC*
Ala	GCA	382	1.12	*trnA-UGC*	Gly	GGA	694	1.59	*trnG-UCC*
Ala	GCG	171	0.5		Gly	GGG	282	0.65	

*SCU, Relative Synonymous Codon Usage.*

### REPEAT ANALYSIS

Four forward, five inverted and eight tandem repeats were identified by REPuter and TRF with a copy size 30 bp or longer (**Table 5**). Most repeats possessed lengths between 30 and 40 bp, and the longest repeat was 52 bp as a forward repeat located the LSC region (*psaA, psaB*, CDS). All tandem repeats were found to be repeated twice in the whole cp genome, and six of these were located in intergenic spacer regions, with the left two located within *ycf2* (CDS) and *rps16* (intron), respectively.

**Table 5:** Repeated sequences in the *A. barbatum* var.* puberulum* chloroplast genome.

Repeat number	Size (bp)	Type	Location	Repeat unit	Region
1	52	F	*psaB*(CDS),* psaA*(CDS)	AGAAAAAGAATTGCAATAGCTAAATGG(A)TGA(G)TGA(C)GCAATATCGGTCAGCCATA	LSC
2	39	F	*ycf3*(CDS), IGS(*trnV-GAC,rps12*)	CAGAACCGTACATGAGATTTTCACCTCATACGGCTCCTC	LSC, Ira
3	33	F	IGS(*psbI, trnS-GCU*), IGS(*psbC, trnS-UGA*)	TAAAC(A)GGAA(G)AGAGAGGGATTCGAACCCTCGG(A)TA	LSC
4	32	F	IGS(*rps15, ycf1*)	TTT(G)TTCT(A)T(A)CTTGATTTAGATTCTCTAATTCAA	SSC
5	39	P	*ycf3*(intron), IGS*(trnV-GAC, rps12*)	CAGAACCGTACATGAGATTTTCACCTCATACGGCTCCTC	LSC, Irb
6	33	P	IGS(*petA, psbJ*), *psbJ*(CDS)	GTAAGAATAAGAACTCAATGGACCTTGCCCCTC	LSC
7	30	P	*trnS-GCU*, *trnS-GGA*	ACGGAAAGAGAGGGATTCGAACCCTCGGTA	LSC
8	31	P	IGS(*trnE-UUC,trnT-GGU*)	TCT(G)ATT(A)TC(A)TTATTTCTATATATTCTAATGAT	LSC
9	30	P	*trnS-UGA, trnS-GGA*	TAC(A)CGAGGGTTCGAATCCCTCTCTT(G)TCCG(A)T	LSC
10	30	T	*rps16* (intron)	TAATAGTATATATAG(×2)	LSC
11	34	T	IGS(*rps16,trnQ-UUU*)	TTTTATTCTATTTATTA(×2)	LSC
12	40	T	IGS*(atpF,atpH)*	GTTATTGTAGGAGTGAAATC(×2)	LSC
13	30	T	IGS*(trnT-GGU,psbD)*	ATAGTCATTATAATG(×2)	LSC
14	36	T	IGS*(rbcL,accD)*	TTTCTATTGTTGTATCCA(×2)	LSC
15	40	T	IGS*(trnP-GGG,psaJ)*	TTTATTATATAAAATATTAA(×2)	LSC
16	42	T	ycf2(CDS)	AGATAATGAACTATTCAAAGA(2)	IRa,b
17	30	T	IGS*(ndhE,ndhG)*	TATTACCTATTATAT(×2)	SSC

### SSR ANALYSIS

Microsatellites in the chloroplast genome are highly informative about genetic diversity and represent a useful tool for population genetics and evolutionary and ecological studies (Powell et al., 1996; Huang and Sun, 2000; Provan et al., 2001). Thus, the SSRs in the cp genome of *A. barbatum* var.* puberulum* were identified for use in future studies. The total number of the mononucleotides (not shorter than 8 bp) was 131, and T represented the highest portion (53.4%) followed by A, C, and G (44.3%, 1.5%, and 0.8%, respectively). The longest poly structure was a 20 T-repeat. In total, 56 dinucleotides were detected throughout the cp genome, and most of them were present as four repetitions (78.6%), e.g., ATATATAT. The combination of AT/TA was the most prevalent dinucleotide (42.9%). Four types of trinucleotide (ATA/ATT/TAT/TTA) were present as multiple A/T nucleotides. Seven tetranucleotides were detected, but no penta- or hexanucleotides (repeated at least three times) were found in the cp genome of *A. barbatum* var.* puberulum*. It can be inferred that the SSR loci contribute to the A+T richness of the cp genome. The longest poly-T and poly-A structures (20-nucleotide repeats and 14-nucleotide repeats, respectively) were located in IGS (*petA-pabJ*), *ycf3*, and IGS (*psaJ-rpl33*).

## DISCUSSION

### *Aconitum* IS HIGHLY TOXIC, AND CHLOROPLAST GENOME IS INFORMATIVE AND REFERABLE FOR MOLECULAR IDENTIFICATION

In recent years, there have been many reports on the improper use of toxic, aconitine-containing plants, which has led to deaths (Poon et al., 2006; Chen et al., 2012). Therefore, *Aconitum* identification is important. With reductions in sequencing costs, cp genomes could be used as super-barcodes in the near future. After sequencing and analyzing the cp genomes of 37 different *Pinus* species, Parks et al. (2009) concluded that cp genomes could be used to improve phylogenetic resolution at lower taxonomic levels and could be thought of as species-level DNA barcodes. Li et al. (2014b) also suggest that complete cp genomes have tremendous potential for the identification of closely related species. *Aconitum* consists of approximately 300 species and its taxonomy has been complex due to the close relationships among different species (Xiao et al., 2005; Jabbour and Renner, 2012). Cp genome regions such as *psb*A-*trn*H have been applied, but it cannot be used to identify all the species of* Aconitum* (He et al., 2010). Hence, whole cp genomes are thought to have the potential in *Aconitum* identification studies. In our study, the successful use of a third-generation sequencing platform provides a new, rapid way to sequence the* Aconitum* cp genomes, which could help to lay the foundation for the molecular identification of *Aconitum* based on its cp genomes.

### CCS READS PROVED TO BE RELIABLE VIA SANGER SEQUENCING VALIDATION

In this study, we demonstrated the feasibility of sequencing a cp genome using the PacBio SMRT third-generation sequencing platform; use of this platform has been shown to be a rapid approach for sequencing small genomes, such as microbial and plasmid genomes (Chin et al., 2013). We evaluated the error-rate of the PacBio *RS* data by comparing its results with those obtained by Sanger sequencing. The CCS reads generated in our study had an error rate of approximately 0.027%, which was lower than the rate reported by Cronn et al. (2008) for Illumina sequencing-by-synthesis technology (0.056%). However, some questions remain regarding the error rate of the PacBio system. The observed raw error rate was 12.86%, which was much higher than that of other platforms, such as Illumina MiSeq and Ion Torrent PGM (Quail et al., 2012). To improve this situation, CCS is thought to be an effective approach. CCS is one of the PacBio *RS* sequencing protocols that performs multiple passes on each molecule that is sequenced. After the application of the necessary QC filters, the result is an error-corrected consensus read with a higher intra-molecular accuracy. This approach results in higher per-base quality and reduced concerns about suspicious results. By generating multiple reads from the same molecule and eliminating errors resulting from single reads, the PacBio system’s inherent error rate can be bypassed. For that reason, in this study, we used the CCS protocol to sequence the *A. barbatum* var.* puberulum* cp genome and obtain high-quality reads. The data presented here show that SMRT sequencing using the CCS strategy is a powerful tool for sequencing cp genomes. In addition, in some extreme situations, we suggest completing genome assembly by combining CCS reads with regular long reads. We believe that this strategy would be an effective way to solve the problems associated with assembling large genomes or genomes that contain special structures.

### THE LONG READS DERIVED FROM PacBio IMPROVE GENOME ASSEMBLY

The long read lengths undoubtedly provide a number of benefits in genome sequencing and assembly. The most obvious benefit is for *de novo* assemblies. Previous studies have shown that, compared with Illumina data, chloroplast genome assembly using the PacBio *RS* sequencer generated longer contigs and fewer unresolved gaps (Ferrarini et al., 2013). In this study, we constructed the draft sequence in a step-by-step manner by extending two seed reads on both the 5′ and 3′ ends until they overlapped at the two IR regions. For all CCS sub-reads, the top BLASTn hit for the seed sequence was selected and used to extend the read. The longer reads (an average of 880 bp) made our assembly and analysis more effective. We encountered no problems mapping the seed sequence reads to the repeat regions of the *A. barbatum* var. *puberulum* cp genome, which are listed in **Table 5**. Even without any other biological or phytological information about the target species, it took less than half an hour to finish the genome assembly step. This strategy is clearly a highly effective and accurate method for obtaining plant cp genomes. In addition, one of the features of the cp genome, the two long IR (regions), is also a valuable target for evaluating the PacBio system. As mentioned above, the comparatively longer CCS reads provided more conveniences on dealing with those special structures.

### ELIMINATING THE PCR AMPLIFICATION STEP SAVES TIME

The SMRT method does not require PCR amplification, which reduces the time required for sequencing. In our study, the sequencing reaction time was 90 min, which streamlined the sequencing process by reducing the overall time in the lab. In addition, eliminating the PCR amplification step alleviated the sequencing bias. In some extreme situations, e.g., AT-rich, GC-rich, and repeat-rich regions, the results are unsatisfactory due to the loss of DNA during amplification (Bashir et al., 2012). The sequencing of unamplified molecules will improve genome assembly and allow the detection unique and informative structures.

## Conflict of Interest Statement

The authors declare that the research was conducted in the absence of any commercial or financial relationships that could be construed as a potential conflict of interest.

## References

[B1] Altschul S. F. Madden T. L. Schaffer A. A. Zhang J. Zhang Z. Miller W. (1997). Gapped BLAST and PSI-BLAST: a new generation of protein database search programs. *Nucleic Acids Res.* 25 3389–3402 10.1093/nar/25.17.3389 9254694 PMC146917

[B2] Bashir A. Klammer A. A. Robins W. P. Chin C.-S. Webster D. Paxinos E. (2012). A hybrid approach for the automated finishing of bacterial genomes. *Nat. Biotechnol.* 30 701–707 10.1038/nbt.2288 22750883 PMC3731737

[B3] Benson G. (1999). Tandem repeats finder: a program to analyze DNA sequences. *Nucleic Acids Res.* 27 573–580 10.1093/nar/27.2.573 9862982 PMC148217

[B4] Chen S. P. Ng S. W. Poon W. T. Lai C. K. Ngan T. M. Tse M. L. (2012). Aconite poisoning over 5 years: a case series in Hong Kong and lessons towards herbal safety. *Drug Saf.* 35 575–587 10.2165/11597470-000000000-00000 22631223

[B5] Chen S. Pang X. Song J. Shi L. Yao H. Han J. (2014). A renaissance in herbal medicine identification: from morphology to DNA. *Biotechnol. Adv.* 32 1237–1244 10.1016/j.biotechadv.2014.07.004 25087935

[B6] Chin C.-S. Alexander D. H. Marks P. Klammer A. A. Drake J. Heiner C. (2013). Nonhybrid, finished microbial genome assemblies from long-read SMRT sequencing data. *Nat. Methods* 10 563–569 10.1038/nmeth.2474 23644548

[B7] Chin C.-S. Sorenson J. Harris J. B. Robins W. P. Charles R. C. Jean-Charles R. R. (2011). The origin of the Haitian cholera outbreak strain. *N. Engl. J. Med.* 364 33–42 10.1056/NEJMoa1012928 21142692 PMC3030187

[B8] Cronn R. Liston A. Parks M. Gernandt D. S. Shen R. Mockler T. (2008). Multiplex sequencing of plant chloroplast genomes using Solexa sequencing-by-synthesis technology. *Nucleic Acids Res.* 36 e122–e122 10.1093/nar/gkn502 18753151 PMC2577356

[B9] Cui L. Veeraraghavan N. Richter A. Wall K. Jansen R. K. Leebens-Mack J. (2006). ChloroplastDB: the chloroplast genome database. *Nucleic Acids Res.* 34 D692–D696 10.1093/nar/gkj055 16381961 PMC1347418

[B10] Daniell H. Datta R. Varma S. Gray S. Lee S.-B. (1998). Containment of herbicide resistance through genetic engineering of the chloroplast genome. *Nat. Biotechnol.* 16 345–348 10.1038/nbt0498-345 9555724 PMC5522713

[B11] Dong W. Xu C. Cheng T. Lin K. Zhou S. (2013). Sequencing angiosperm plastid genomes made easy: a complete set of universal primers and a case study on the phylogeny of Saxifragales. *Genome Biol. Evol.* 5 989–997 10.1093/gbe/evt063 23595020 PMC3673619

[B12] Eid J. Fehr A. Gray J. Luong K. Lyle J. Otto G. (2009). Real-time DNA sequencing from single polymerase molecules. *Science* 323 133–138 10.1126/science.1162986 19023044

[B13] Ferrarini M. Moretto M. Ward J. A. Surbanovski N. Stevanovic V. Giongo L. (2013). An evaluation of the PacBio RS platform for sequencing and *de novo* assembly of a chloroplast genome. *BMC Genomics* 14:670 10.1186/1471-2164-14-670 PMC3853357 24083400

[B14] Gilles A. Meglécz E. Pech N. Ferreira S. Malausa T. Martin J.-F. (2011). Accuracy and quality assessment of 454 GS-FLX Titanium pyrosequencing. *BMC Genomics* 12:245 10.1186/1471-2164-12-245 PMC3116506 21592414

[B15] He J. Wong K.-L. Shaw P.-C. Wang H. Li D.-Z. (2010). Identification of the medicinal plants in *Aconitum* L. by DNA barcoding technique. *Planta Med.* 76 1622–1628 10.1055/s-0029-1240967 20217641

[B16] Hiratsuka J. Shimada H. Whittier R. Ishibashi T. Sakamoto M. Mori M. (1989). The complete sequence of the rice (*Oryza sativa*) chloroplast genome: intermolecular recombination between distinct tRNA genes accounts for a major plastid DNA inversion during the evolution of the cereals. *Mol. Gen. Genet.* 217 185–194 10.1007/BF02464880 2770692

[B17] Huang J. Sun M. (2000). Genetic diversity and relationships of sweet potato and its wild relatives in *Ipomoea* series batatas (Convolvulaceae) as revealed by inter-simple sequence repeat (ISSR) and restriction analysis of chloroplast DNA. *Theor. Appl. Genet.* 100 1050–1060 10.1007/s001220051386

[B18] Huang X. Madan A. (1999). CAP3: A DNA sequence assembly program. *Genome Res.* 9 868–877 10.1101/gr.9.9.868 10508846 PMC310812

[B19] Jabbour F. Renner S. S. (2012). A phylogeny of Delphinieae (Ranunculaceae) shows that *Aconitum* is nested within *Delphinium* and that Late Miocene transitions to long life cycles in the Himalayas and Southwest China coincide with bursts in diversification. *Mol. Phylogenet. Evol.* 62 928–942 10.1016/j.ympev.2011.12.005 22182994

[B20] Johansson J. T. (1995). A revised chloroplast DNA phylogeny of the Ranunculaceae, in systematics and evolution of the Ranunculiflorae. *Springer* 9 253–261.

[B21] Jordan W. C. Courtney M. W. Neigel J. E. (1996). Low levels of intraspecific genetic variation at a rapidly evolving chloroplast DNA locus in North American duckweeds (Lemnaceae). *Am. J. Bot.* 83 430–439 10.2307/2446212

[B22] Kircher M. Heyn P. Kelso J. (2011). Addressing challenges in the production and analysis of illumina sequencing data. *BMC Genomics* 12:382 10.1186/1471-2164-12-382 PMC3163567 21801405

[B23] Kurtz S. Choudhuri J. V. Ohlebusch E. Schleiermacher C. Stoye J. Giegerich R. (2001). REPuter: the manifold applications of repeat analysis on a genomic scale. *Nucleic Acids Res.* 29 4633–4642 10.1093/nar/29.22.4633 11713313 PMC92531

[B24] Li H. Durbin R. (2010). Fast and accurate long-read alignment with Burrows-Wheeler transform. *Bioinformatics* 26 589–595 10.1093/bioinformatics/btp698 20080505 PMC2828108

[B25] Li Q. Li Y. Song J. Xu H. Xu J. Zhu Y. (2014a). High-accuracy *de novo* assembly and SNP detection of chloroplast genomes using a SMRT circular consensus sequencing strategy. *New Phytol.* 204 1041–1049 10.1111/nph.12966 25103547

[B26] Li X. Hu Z. Lin X. Li Q. Gao H. Luo G. (2012). High-throughput pyrosequencing of the complete chloroplast genome of Magnolia officinalis and its application in species identification. *Acta Pharm. Sin.* 47 124–130. 22493817

[B27] Li X. Yang Y. Henry R. J. Rossetto M. Wang Y. Chen S. (2014b). Plant DNA barcoding: from gene to genome. *Biol. Rev. Camb. Philos. Soc.* 10.1111/brv.12104 [Epub ahead of print]. 24666563

[B28] Lohse M. Drechsel O. Bock R. (2007). OrganellarGenomeDRAW (OGDRAW): a tool for the easy generation of high-quality custom graphical maps of plastid and mitochondrial genomes. *Curr. Genet.* 52 267–274 10.1007/s00294-007-0161-y 17957369

[B29] Mardis E. R. (2008a). The impact of next-generation sequencing technology on genetics. *Trends Genet.* 24 133–141 10.1016/j.tig.2007.12.007 18262675 PMC2680276

[B30] Mardis E. R. (2008b). Next-generation DNA sequencing methods. *Annu. Rev. Genomics Hum. Genet.* 9 387–402 10.1146/annurev.genom.9.081307.164359 18576944

[B31] McPherson H. Van Der Merwe M. Delaney S. K. Edwards M. A. Henry R. J. Mcintosh E. (2013). Capturing chloroplast variation for molecular ecology studies: a simple next generation sequencing approach applied to a rainforest tree. *BMC Ecol.* 13:8 10.1186/1472-6785-13-8 PMC3605380 23497206

[B32] Metzker M. L. (2009). Sequencing technologies—the next generation. *Nat. Rev. Genet.* 11 31–46 10.1038/nrg2626 19997069

[B33] Millen R. S. Olmstead R. G. Adams K. L. Palmer J. D. Lao N. T. Heggie L. (2001). Many parallel losses of infA from chloroplast DNA during angiosperm evolution with multiple independent transfers to the nucleus. *Plant Cell* 13 645–658 10.1105/tpc.13.3.645 11251102 PMC135507

[B34] Moore M. J. Soltis P. S. Bell C. D. Burleigh J. G. Soltis D. E. (2010). Phylogenetic analysis of 83 plastid genes further resolves the early diversification of eudicots. *Proc. Natl. Acad. Sci. U.S.A.* 107 4623–4628 10.1073/pnas.0907801107 20176954 PMC2842043

[B35] Nie X. Lv S. Zhang Y. Du X. Wang L. Biradar S. S. (2012). Complete chloroplast genome sequence of a major invasive species, crofton weed (*Ageratina adenophora*). *PLoS ONE* 7:e36869 10.1371/journal.pone.0036869 PMC3350484 22606302

[B36] Pan I.-C. Liao D.-C. Wu F.-H. Daniell H. Singh N. D. Chang C. (2012). Complete chloroplast genome sequence of an orchid model plant candidate: *Erycina pusilla* apply in tropical *Oncidium* breeding. *PLoS ONE* 7:e34738 10.1371/journal.pone.0034738 PMC3319614 22496851

[B37] Parks M. Cronn R. Liston A. (2009). Increasing phylogenetic resolution at low taxonomic levels using massively parallel sequencing of chloroplast genomes. *BMC Biol.* 7:84 10.1186/1741-7007-7-84 PMC2793254 19954512

[B38] Pfannschmidt T. Nilsson A. Allen J. F. (1999). Photosynthetic control of chloroplast gene expression. *Nature* 397 625–628 10.1038/17624

[B39] Poon W. T. Lai C. K. Ching C. K. Tse K. Y. So Y. C. Chan Y. C. (2006). Aconite poisoning in camouflage. *Hong Kong Med. J.* 12 456–459. 17148799

[B40] Powell W. Machray G. C. Provan J. (1996). Polymorphism revealed by simple sequence repeats. *Trends Plant Sci.* 1 215–222 10.1016/1360-1385(96)86898-1

[B41] Provan J. Powell W. Hollingsworth P. M. (2001). Chloroplast microsatellites: new tools for studies in plant ecology and evolution. *Trends Ecol. Evol. (Amst.)* 16 142–147 10.1016/S0169-5347(00)02097-8 11179578

[B42] Qian J. Song J. Gao H. Zhu Y. Xu J. Pang X. (2013). The complete chloroplast genome sequence of the medicinal plant *Salvia miltiorrhiza*. *PLoS ONE* 8:e57607 10.1371/journal.pone.0057607 PMC3584094 23460883

[B43] Quail M. A. Smith M. Coupland P. Otto T. D. Harris S. R. Connor T. R. (2012). A tale of three next generation sequencing platforms: comparison of ion torrent, pacific biosciences and illumina miSeq sequencers. *BMC Genomics* 13:341 10.1186/1471-2164-13-341 PMC3431227 22827831

[B44] Rasko D. A. Webster D. R. Sahl J. W. Bashir A. Boisen N. Scheutz F. (2011). Origins of the E. coli strain causing an outbreak of hemolytic–uremic syndrome in Germany. *N. Engl. J. Med.* 365 709–717 10.1056/NEJMoa1106920 21793740 PMC3168948

[B45] Roberts R. J. Carneiro M. O. Schatz M. C. (2013). The advantages of SMRT sequencing. *Genome Biol.* 14 405 10.1186/gb-2013-14-6-405 PMC3953343 23822731

[B46] Rusk N. (2009). Cheap third-generation sequencing. *Nat. Methods* 6 244–244 10.1038/nmeth0409-244a 19885970

[B47] Sato S. Nakamura Y. Kaneko T. Asamizu E. Tabata S. (1999). Complete structure of the chloroplast genome of *Arabidopsis thaliana*. *DNA Res.* 6 283–290 10.1093/dnares/6.5.283 10574454

[B48] Schadt E. E. Turner S. Kasarskis A. (2010). A window into third-generation sequencing. *Hum. Mol. Genet.* 19 R227–R240 10.1093/hmg/ddq416 20858600

[B49] Schattner P. Brooks A. N. Lowe T. M. (2005). The tRNAscan-SE, snoscan and snoGPS web servers for the detection of tRNAs and snoRNAs. *Nucleic Acids Res.* 33 W686–W689 10.1093/nar/gki366 15980563 PMC1160127

[B50] Shinozaki K. Ohme M. Tanaka M. Wakasugi T. Hayashida N. Matsubayashi T. (1986). The complete nucleotide sequence of the tobacco chloroplast genome: its gene organization and expression. *EMBO J.* 5 2043–2049. 16453699 10.1002/j.1460-2075.1986.tb04464.x PMC1167080

[B51] Sugiura M. (1992). The chloroplast genome. *Plant Mol. Biol.* 19 149–168 10.1007/BF00015612 1600166

[B52] Tamura K. Peterson D. Peterson N. Stecher G. Nei M. Kumar S. (2011). MEGA5: molecular evolutionary genetics analysis using maximum likelihood, evolutionary distance, and maximum parsimony methods. *Mol. Biol. Evol.* 28 2731–2739 10.1093/molbev/msr121 21546353 PMC3203626

[B53] Uthaipaisanwong P. Chanprasert J. Shearman J. Sangsrakru D. Yoocha T. Jomchai N. (2012). Characterization of the chloroplast genome sequence of oil palm (*Elaeis guineensis Jacq*.). *Gene* 500 172–180 10.1016/j.gene.2012.03.061 22487870

[B54] Wu J. Liu B. Cheng F. Ramchiary N. Choi S. R. Lim Y. P. (2012). Sequencing of chloroplast genome using whole cellular DNA and Solexa sequencing technology. *Front. Plant Sci.* 3:243 10.3389/fpls.2012.00243 PMC3492724 23162558

[B55] Wyman S. K. Jansen R. K. Boore J. L. (2004). Automatic annotation of organellar genomes with DOGMA. *Bioinformatics* 20 3252–3255 10.1093/bioinformatics/bth352 15180927

[B56] Xiao P. Wang F. Gao F. Yan L. Chen D. Liu Y. (2005). A pharmacophylogenetic study of *Aconitum* L.(*Ranunculaceae)* from China. *Acta Phytotaxon. Sin.* 44 1–46 10.1360/aps050046

[B57] Yi D.-K. Kim K.-J. (2012). Complete chloroplast genome sequences of important oilseed crop *Sesamum indicum* L. *PLoS ONE* 7:e35872 10.1371/journal.pone.0035872 PMC3351433 22606240

[B58] Zhang T. Fang Y. Wang X. Deng X. Zhang X. Hu S. (2012). The complete chloroplast and mitochondrial genome sequences of *Boea hygrometrica*: insights into the evolution of plant organellar genomes. *PLoS ONE* 7:e30531 10.1371/journal.pone.0030531 PMC3264610 22291979

